# Optomechanical assessment of photorefractive corneal cross-linking via optical coherence elastography

**DOI:** 10.3389/fbioe.2023.1272097

**Published:** 2023-11-13

**Authors:** Matteo Frigelli, Philippe Büchler, Sabine Kling

**Affiliations:** ^1^ Computational Bioengineering Group, ARTORG Center for Biomedical Engineering Research, University of Bern, Bern, Switzerland; ^2^ Institute for Biomedical Engineering, ITET Department, ETH Zürich, Zürich, Switzerland

**Keywords:** optical coherence elastography, corneal cross-linking, optomechanical changes, accelerated CXL, ambient pressure modulation, corneal biomechanics. (Min.5-Max. 8

## Abstract

**Purpose:** Corneal cross-linking (CXL) has recently been used with promising results to positively affect corneal refractive power in the treatment of hyperopia and mild myopia. However, understanding and predicting the optomechanical changes induced by this procedure are challenging.

**Methods:** We applied ambient pressure modulation based optical coherence elastography (OCE) to quantify the refractive and mechanical effects of patterned CXL and their relationship to energy delivered during the treatment on porcine corneas. Three different patterned treatments were performed, designed according to Zernike polynomial functions (*circle*, *astigmatism*, *coma*). In addition, three different irradiation protocols were analyzed: standard Dresden CXL (fluence of 5.4 J/cm^2^), accelerated CXL (fluence of 5.4 J/cm^2^), and high-fluence CXL (fluence of 16.2 J/cm^2^). The axial strain distribution in the stroma induced by ocular inflation (Δp = 30 mmHg) was quantified, maps of the anterior sagittal curvature were constructed and cylindrical refraction was assessed.

**Results:** Thirty minutes after CXL, there was a statistically significant increase in axial strain amplitude (*p* < 0.050) and a reduction in sagittal curvature (*p* < 0.050) in the regions treated with all irradiation patterns compared to the non-irradiated ones. Thirty-6 hours later, the non-irradiated regions showed compressive strains, while the axial strain in the CXL-treated regions was close to zero, and the reduction in sagittal curvature observed 30 minutes after the treatment was maintained. The Dresden CXL and accelerated CXL produced comparable amounts of stiffening and refractive changes (*p* = 0.856), while high-fluence CXL produced the strongest response in terms of axial strain (6.9‰ ± 1.9‰) and refractive correction (3.4 ± 0.9 D). Tripling the energy administered during CXL resulted in a 2.4-fold increase in the resulting refractive correction.

**Conclusion:** OCE showed that refractive changes and alterations in corneal biomechanics are directly related. A patient-specific selection of both, the administered UV fluence and the irradiation pattern during CXL is promising to allow customized photorefractive corrections in the future.

## Introduction

Corneal cross-linking (CXL) is a proven treatment to stop the progression of keratoconus and corneal ectasia, by creating cross-links in the extracellular matrix (Spoerl et al., 1998; Hayes et al., 2011). The ultimate goal of this procedure is to photochemically stiffen the cornea by modifying its biomechanical and biochemical properties. In several cases, CXL has been shown to reduce refractive errors by flattening the corneal topography of ectatic corneas (Hersh et al., 2011). As the cornea is constantly subjected to homogeneous loading by the intraocular pressure (IOP), a local modification of the tissue stiffness will induce a change in its shape and, consequently, in its refractive power. The potential of CXL to favorably alter corneal refractive power has recently been explored for the correction of hyperopia and low myopia in healthy individuals with promising results (Sachdev et al., 2020; Stodulka et al., 2020). The clinical interest in CXL as a treatment for refractive correction is growing, due to the minimally invasive nature of this procedure. It has been suggested that even asymmetric refractive corrections could be achieved by spatially limiting the region of CXL treatment (Seiler et al., 2016). However, to develop a more refined and better predictable protocol for refractive corrections by CXL, a better understanding of the relationship between the degree of tissue stiffening and the resulting refractive change is required.

For the CXL process to occur, riboflavin must be irradiated within the cornea using UVA light in an oxygenated environment in order to generate reactive oxygen species and trigger photodynamic reactions, which in turn promote the formation of new cross-links within the extracellular matrix (Hayes et al., 2011; Zhang et al., 2011). Originally, the treatment delivered UVA light at 3 mW/cm^2^ for 30 min (Wollensak et al., 2003), but over the years various protocols have been developed to speed up the procedure by delivering a higher amount of energy in a shorter period of time according to the Bunsen-Roscoe law of reciprocity. At the same time, it has been shown that sufficient oxygen diffusion into the stroma (Abrishamchi et al., 2021) must be ensured, otherwise the overall efficacy of the treatment decreases in terms of biomechanical stiffening (Richoz et al., 2013). A shortened CXL protocol improves patient comfort, reduces the risk of infection, and could alleviate hospital waiting times (Wernli et al., 2013). Numerous studies have investigated the relationship between the amount of energy administered during CXL and the resulting stromal stiffening. Results from studies (Liu et al., 2020; Boschetti et al., 2021) confirmed that the stiffening effect increases when a higher irradiation fluence is administered. However, to date, the relationship between irradiation energy, mechanical stiffening, and the induced refractive correction remains unclear.

Another challenge is the *in vivo* assessment of the biomechanical properties of the cornea, and how they change in patients during CXL. Air-puff tonometry (Hong et al., 2013) has been evaluated as a technique to estimate corneal stiffness in patients. While widely used in clinical practice (Ambrósio et al., 2017; Esporcatte et al., 2020), this technique’s accuracy in assessing corneal biomechanics has been questioned due to its heavy reliance on IOP and eye geometry (Kling and Marcos, 2013). In addition, the air-puff generated during the measurement bends the cornea inward, causing collagen fibers in the anterior cornea to relax, no longer supporting any load (Ariza-Gracia et al., 2015). Hence, this technique tests a condition that is not representative of the physiologic state. Furthermore, it is not possible to measure stiffness in different areas of the cornea with air pressure tonometry, as only global macroscopic measurements can be obtained (Vinciguerra et al., 2016).

More recently, Brillouin microscopy (Scarcelli and Yun, 2007) and Optical Coherence Elastography (OCE) (Kling et al., 2020) have been introduced as promising tools to quantify corneal mechanical properties with high spatial resolution. The former measures the non-linear scattering originating from a localized volume of tissue when irradiated with a laser beam (Scarcelli et al., 2013). The resulting optical shift is related to the tissue’s longitudinal elastic module, and can therefore be used as an indirect measure of corneal stiffness. A depth-dependent increase in Brillouin modulus has been described in *ex vivo* porcine corneas after CXL, with or without epithelial debridement (Scarcelli et al., 2013). In an *ex vivo* setting, Brillouin microscopy has also been shown to correctly visualize CXL-treated areas when UV irradiation was limited to specific regions of the cornea, indicating local changes in longitudinal mechanical moduli (Kwok et al., 2016). When applied *in vivo*, the longitudinal Brillouin modulus captured significant differences between keratoconus patients and healthy controls. However, *in vivo* Brillouin measurements showed no statistical differences after CXL compared to untreated patients (Shao et al., 2018).

OCE, on the other hand, quantifies the deformation between two consecutive optical coherence tomography scans (OCT) in response to a mechanical stimulus. In the past, phase-sensitive processing has been shown to quantify displacement dynamics and axial strain distribution (Kennedy et al., 2012; Larin and Sampson, 2017) in response to a micro air-puff, corneal applanation, or ambient pressure modulation (Schmitt, 1998; Curatolo et al., 2020; Kling et al., 2020). Several other non-contact OCE techniques have been recently proposed, exploiting sound excitation (Kling et al., 2014; McAuley et al., 2022), ultrasounds (Zvietcovich et al., 2020), and the heartbeat (Nair et al., 2021) to stimulate the tissue. When applied to *ex vivo* porcine corneas, noncontact OCE showed an increase in elastic anisotropy and in Young’s modulus after CXL treatment (Singh et al., 2017). Recently, OCE was able to reveal an altered strain distribution in *ex vivo* rat eyes after CXL treatment (Kling, 2020) that was restricted to the regions exposed to UV irradiation. When applied to *ex vivo* human corneas, OCE measured the changes in the anisotropic elastic properties induced by CXL (Kirby et al., 2023). OCE has also been used *in vivo*: De Stefano and others (De Stefano et al., 2020) demonstrated for the first time on human eyes the effectiveness of OCE in detecting the depth-dependent biomechanical abnormalities in keratoconus by applanation of the cornea with a lens. Zvietcovich et al. assessed differences induced by localized CXL treatment on *in vivo* rabbit corneas exploiting a confocal air-coupled OCE setup (Zvietcovich et al., 2022). More recently, noncontact OCE devices have been tested for *in vivo* evaluation of corneal biomechanics by performing clinical studies on humans, applying either a dynamic modulation of ambient pressure within a physiologic range (Kling, 2021), a mechanical tissue stimulation with micro air-pulse (Lan et al., 2021) or Rayleigh type elastic waves (Ramier et al., 2020).

For CXL to be employed in everyday clinical practice as a treatment for refractive errors, it is crucial to understand whether there is a direct relation between the irradiation pattern, the induced degree of tissue stiffening and the resulting curvature change, in order to fully control and predict the location and extent of the resultant refractive correction. Our hypothesis is that the curvature of the cornea can be individually corrected through localized stiffening via patterned corneal CXL. We evaluate this hypothesis by applying ambient-pressure modulation based OCE to quantify the *ex vivo* mechanical and refractive changes induced by localized CXL with distinct irradiation patterns and at different UV fluences. Furthermore, we quantify the stability of the induced optomechanical effects by assessing the outcome 30 min and 36 h post-op.

## Materials and methods

### OCT system

The study was performed on a previously described spectral domain OCT system (Kling et al., 2020), which operates at a central wavelength of 877.8 nm, a bandwidth of 62.5 nm and an output power of 1.62 mW. At each measurement step, a volumetric C-scan of a 6 × 6 mm region of the central cornea was acquired, consisting of a stack of 100 B-scans (2D tomographic images) with an axial and lateral resolution of 4.5 μm (in air) and 12 μm, respectively.

### Strain computation

For the calculation of the axial strain, two OCT C-scans were compared to assess the mechanical deformation that occurred in the time between the first and the second scan. For this purpose, a phase-sensitive deformation tracking algorithm (Kling et al., 2020) was implemented in customized routines written in MATLAB (Massachusetts: The MathWorks Inc., 2019). Briefly, phase differences corresponding to the angle of С(z, x) were calculated by amplitude-weighted complex cross-correlation, using the formula:

$$C\left(z,x\right)=\sum_{j=-v_{z}}^{v_{z}}\sum_{k=-v_{x}}^{v_{x}}A_{1}\left(z+j,x+k\right)\;\cdot \;A_{2}^{*}\left(z+j,x+k\right)$$ (1)

Where A (z, x) represents the complex OCT interference signal recorded at the axial position z and lateral position x [m], of either the first (A_1_) or second (A_2_) OCT scans. *v*_z_ = 3 and *v*_x_ = 3 [pixels] is the size of the applied phase-processing windows.

The following equations (Matveyev et al., 2018; Za et al., 2016; Zykov et al., 2023) were used to obtain the pixel-wise strain in the direction of the optical axis z [m], ${ɛ}_{zz}$ [-]:

$$U\left(z,x\right)=\frac{\lambda_{mean}*∠C\left(z,x\right)}{4\pi n}$$ (2)

where U (z, x) [m] is the axial displacement, λ_mean_ = 877.8 nm is the central wavelength, and n = 1.375 [-] is the refractive index of the cornea. It follows that the strain in the axial direction is given by the angle of a second complex cross-correlation ∠R (z, x) [rad] via:

$$\varepsilon_{zz}\left(z,x\right)=\frac{dU}{dz}=\frac{\lambda_{mean}*∠R\left(z,x\right)}{4\pi n\delta }$$ (3)

where $R\left(z,x\right)=\sum_{j=-w_{z}}^{w_{z}}\sum_{k=-w_{x}}^{w_{x}}C\left(z+j,x+k\right)\;\cdot \;C^{*}\left(z+1+j,x+k\right)$ , δ = 4.48 μm is the axial sampling unit. *w*_z_ = 3 and *w*_x_ = 3 [pixels] is the size of the applied phase-processing windows. Accordingly, the resulting strain map had an axial and lateral resolutions of 39 × 168 μm, respectively.

### Anterior surface segmentation

In each 2D image, the anterior surface was segmented using an in-house MATLAB script. Starting from a manual selection of the apex of the cornea, the algorithm finds the brightest pixel for each A-scan and classifies it as anterior surface. The algorithm then corrects spatial outliers using nearest-neighbor interpolation, replacing incorrectly classified pixels by interpolating their positions starting with surrounding pixels at the apex, which have been manually identified.

With this segmented anterior surface, the strain image was transformed to a flat surface for subsequent en-face visualization. The anterior segmentation was also used to determine the refractive power of the cornea.

### Refractive power analysis

The anterior surfaces obtained from the segmented 2D images were combined to form a 3D point cloud, which was translated to have the apex at the origin of the Cartesian coordinate system. In addition, the point cloud was rotated to have the apex outer normal parallel to the *z*-axis. The resulting surface was interpolated using the Matlab command *griddata* to calculate the local sagittal curvature K of the anterior cornea:

$$K=\frac{\left(n-1\right)}{R_{a}}$$ (4)

where n = 1.3375 is the refractive index and R_a_ is the local sagittal radius of the anterior surface. Similar to the strain image, the sagittal power map is presented as en-face view.

Next, a series of orthogonal Zernike polynomials (von, 1934) (order = 9) was applied to fit the 3D point cloud representing the corneal anterior surface within an optical zone of radius 3mm, and the residual error was minimized to obtain the best fit. The Zernike coefficients were used to quantitatively analyze the surface aberrations, i.e., the wavefront error, which is a measure of the CXL-induced refractive change. In addition, three measures of corneal cylindrical and spherical power were derived from the Zernike coefficients according to the following formulas (Thibos et al., 2004):

$$M=\frac{-c_{2}^{0}4\sqrt{3}}{R_{p}^{2}}\;\;Cyl=-2\;\sqrt{J_{0}^{2}+J_{45}^{2}}J_{0}=\frac{-c_{2}^{2}2\sqrt{6}}{R_{p}^{2}}\;\;\Phi =\frac{1}{2}\tan^{-1}\left(\frac{J_{45}}{J_{0}}\right)\;J_{45}=\frac{-c_{-2}^{2}2\sqrt{6}}{R_{p}^{2}}\;\;Sph=M-\frac{Cyl}{2}$$ (5)

Where $c_{n}^{m}$ is the *n*th Zernike coefficient of frequency m and R_p_ is the radius of the pupil (usually the iris is considered). The optical system can be described by the ordinary cylinder with positive power J at axis α, denoted by Jα, and of the spherical equivalent power of the lens, denoted by M. Additionally, the cylindrical power, *Cyl*, the angle of astigmatism, *Φ*, and the spherical power, *Sph*, can be derived. It should be noted that a mean value of the iris radius for porcine eyes (Rp = 7 mm) was considered according to previously published topographic findings (Sanchez et al., 2011).

### Patterned CXL protocols

A total of fifteen freshly-enucleated porcine eyes were bought from the local slaughterhouse (Micarna Shop, 1784 Courtepin, Switzerland) and tested between 4 and 48 h post-mortem. For CXL treatment, the epithelium was carefully removed using a blunt knife. A 0.1% riboflavin (Streuli Pharma AG, Switzerland) in phosphate buffered saline (PBS) solution was administered every 5 min for 20 min before UVA irradiation with a 365 nm lamp (LED UV Curing System, Thorlabs, New Jersey) to ensure that the cornea sufficiently absorbed the photosensitizer and throughout the irradiation period to avoid tissue dehydration.

Each eye was stored in a refrigerator at 4 °C before undergoing patterned CXL treatment within 12 h of collection. Corneal biomechanical and refractive changes were assessed at three timepoints during the procedure: preoperative, 30 min post-op, and 36 h postoperative. For the purpose of this study, the intraocular pressure was assumed to be the same across all samples.

The sample cohort was divided into three subgroups: 9 eyes were assigned to the Dresden group (group A), 3 to the accelerated CXL (group B) group and 3 to the high-fluence group (group C). Group A was subjected to a standard Dresden protocol for CXL treatment (365nm, 3 mW/cm^2^ for 30 min, resulting in a fluence of 5.4 J/cm^2^) (Wollensak et al., 2003), with the difference that UV irradiation was limited to specific regions on the cornea defined by three different irradiation masks (n = 3 per mask). The eyes of groups B and C were both treated with an *astigmatism* pattern but different UV irradiation regimens. Group B was subjected to an accelerated CXL protocol, irradiating for 10 min with an irradiance of 9 mW/cm^2^ (fluence of 5.4 J/cm^2^), while group C was irradiated for 30 min with 9 mW/cm^2^ (fluence of 16.2 J/cm^2^).

**FIGURE 1:**
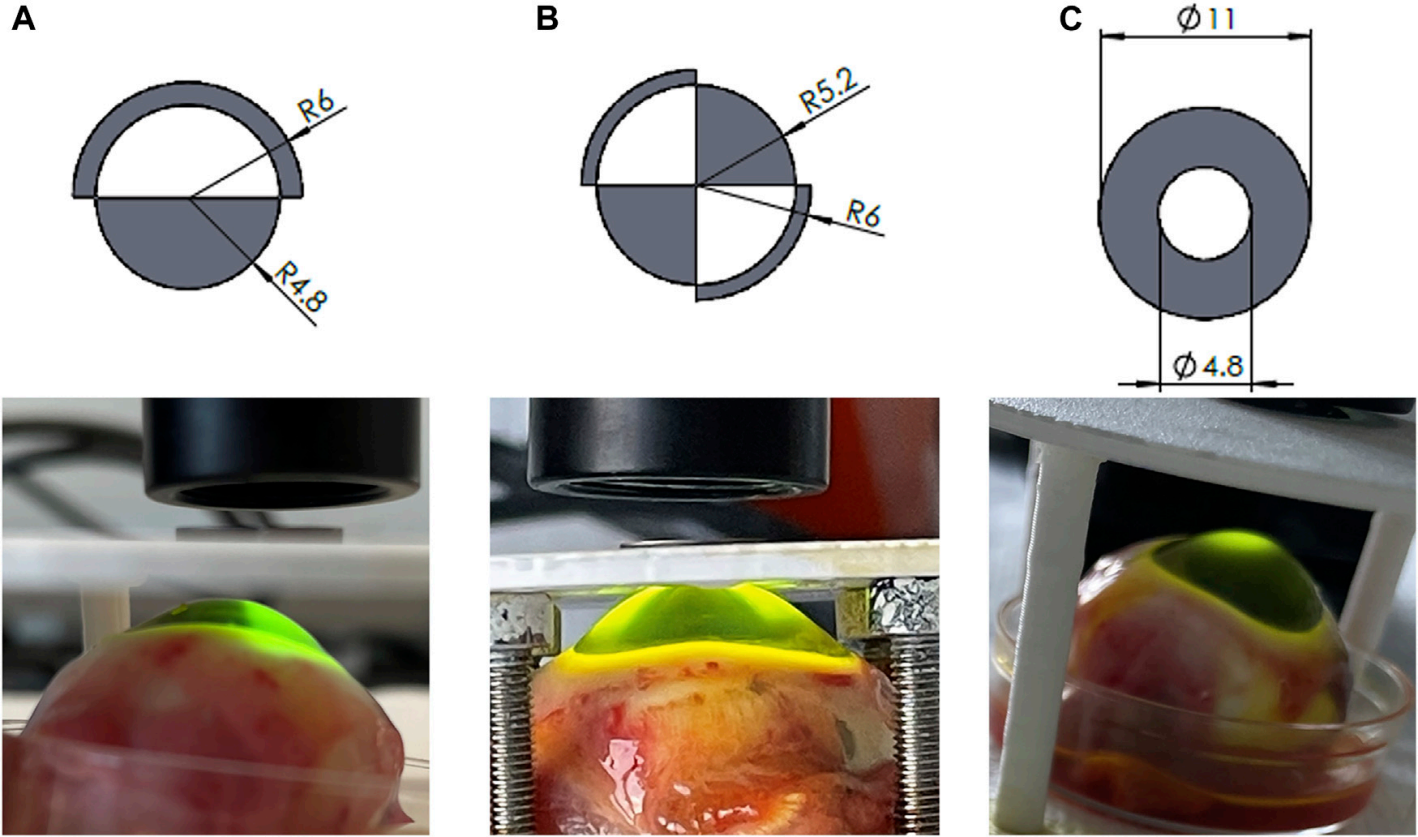
Zernike-derived masks applied to spatially confine UV irradiation representing **(A)** coma, **(B)** astigmatism, **(C)** circle patterns. Top panel: masks design; bottom panel: patterned UV irradiation.

To achieve different CXL patterns, we designed three steel masks taking inspiration form the Zernike functions: (Figures 1A–C): i) the first mask allowed irradiation of half of the cornea surface (*coma*), ii) the second allowed irradiation of two opposite quarters (*astigmatism*) and iii) the third mask allowed only the irradiation of the central cornea within a 4.8 mm diameter (*circle*). The masks were defined by taking the sign of the first Zernike polynomials (up to the 4th order) on a 12 mm diameter circle. The corresponding coordinates were read into SolidWorks (Dassault Systèmes, United States) and used as templates for the 3D models. Finally, the mask CADs were sent to a company specialized in laser cutting (Felastec GmbH Feinstlasertechnik, 3,800 Unterseen, Switzerland) which produce them with a stainless steel sheet of 1 mm thickness. During UV irradiation, the masks were placed between the cornea and the UV source irradiate the appropriate areas of the cornea, localizing CXL treatment.

After completion of the CXL treatment, the eyes were stored for 36–48 h in a minimum essential medium (MeM) containing 5% Dextran in a refrigerator at 4 °C to stabilize tissue hydration.

#### OCE assessment

**FIGURE 2:**
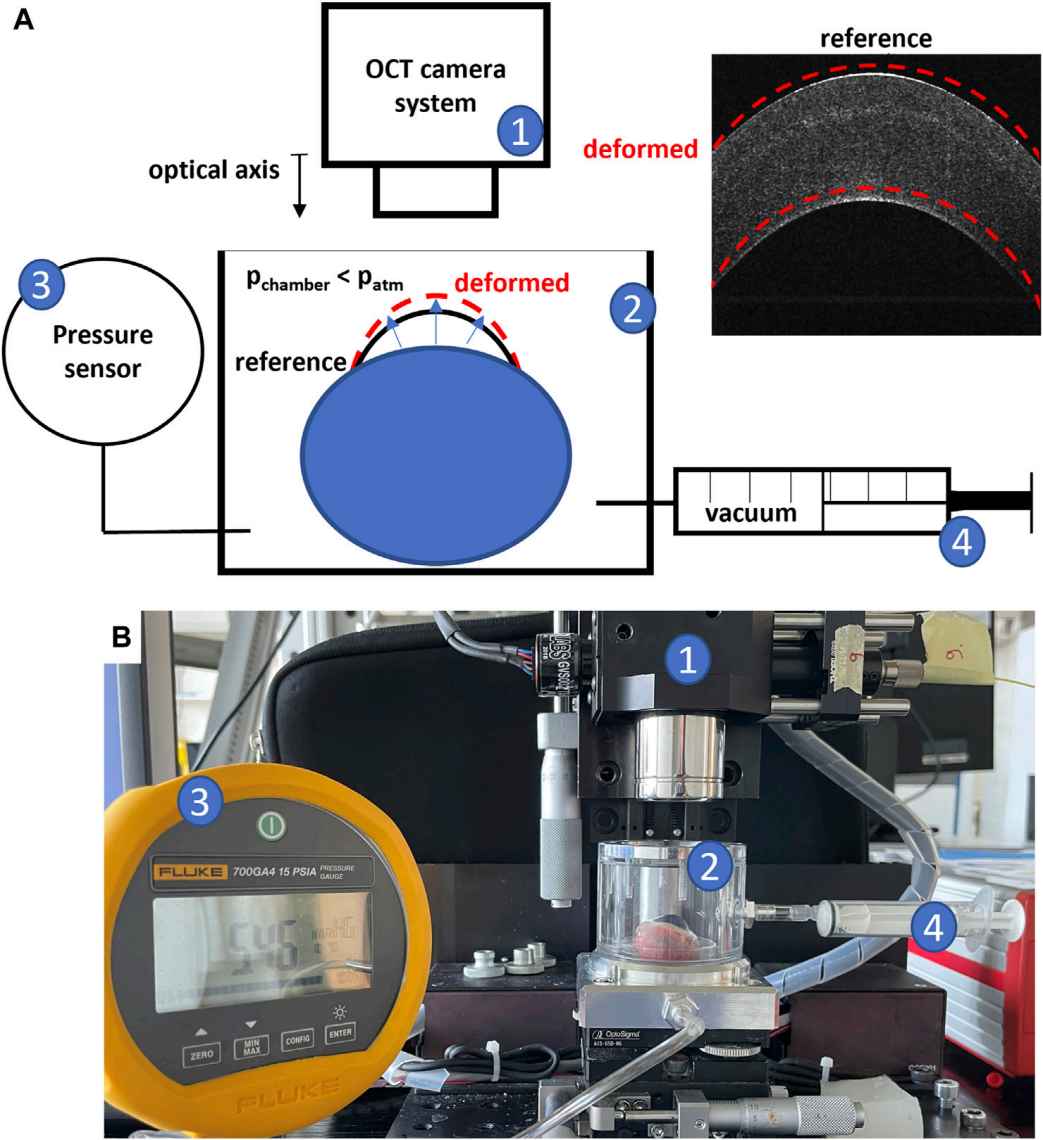
OCE Experimental setup; **(A)** schematic representation; **(B)** picture of the setup; 1) OCT camera system; 2) pressure chamber in which the eye globe is placed; 3) pressure sensor; 4) syringe to modulate pressure in the chamber.

The biomechanical and refractive assessment was performed using the OCE setup shown in Figure 2. It consists of a transparent pressure chamber placed under a spectral domain OCT system. The chamber is connected to both a pressure sensor (700G Series Pressure Gauge, Fluke, Everett, Washington) and an empty 5-mL syringe for controlled modulation of ambient pressure. A non-contact inflation experiment was performed by placing the entire eyeball within the pressure chamber and applying a mild vacuum using the syringe (Δp = 30 mmHg, achieved by withdrawing 3 mL of air from the chamber). The slight vacuum causes an increased stress on the ocular wall, which in a normal material leads to an axial compression that is measurable via OCE. For a single OCE measurement (repeated at different time points during the treatment), two volumetric scans were acquired, before and after applying the vacuum. A single OCE measurement took approximately 1 min. A total of three OCE measurements were acquired: i) after instillation of riboflavin, ii) 30 min after patterned UV irradiation, and iii) 36–48 h later. From each OCE dataset, both the pixel-wise en-face axial strain and sagittal curvature map were derived. In addition, the differences between the three measurement points were evaluated in terms of induced refractive correction.

#### Statistical methods

Statistical analyses were performed in GraphPad Prism 8.0.1 (Graph-Pad Software Inc., La Jolla, Calif). Continuous variables were expressed as means (± standard deviation) or medians (Q1-Q3).

Irradiated and non-irradiated regions were compared in terms of axial strains and sagittal curvatures. Student’s t-test or paired t-tests were performed to test for statistically significant differences between irradiated and non-irradiated areas or between the same regions at different treatment phases. One-tailed ANOVA was performed to test for statistically significant differences between different CXL patterns (*coma, astigmatism, circle*) or between different CXL protocols (Groups A, B and C). Differences were considered statistically significant for *p*-values<0.05.

## Results

### The effect of different irradiation patterns

**FIGURE 3:**
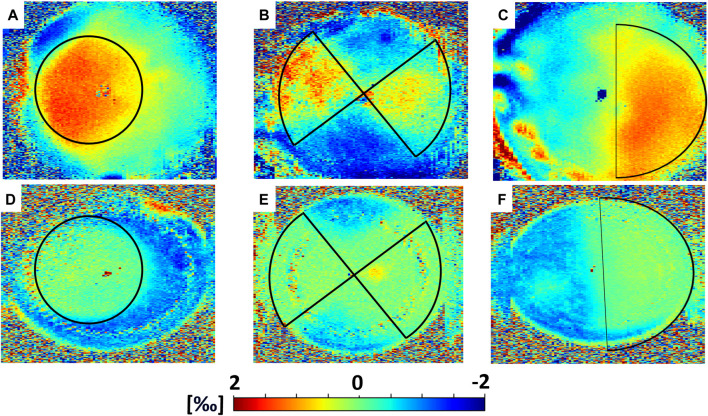
Mechanical effects induced by CXL in Group A (Dresden Protocol), averaged on the first 350 µm. Top panel: axial strain as computed 30 min after CXL for **(A)** *circle*, **(B)** *astigmatism* and **(C)** *coma* irradiation patterns. Bottom panel: axial strain as computed 36 h after CXL for **(D)** *circle*, **(E)** *astigmatism* and **(F)** *coma* irradiation patterns.

**FIGURE 4:**
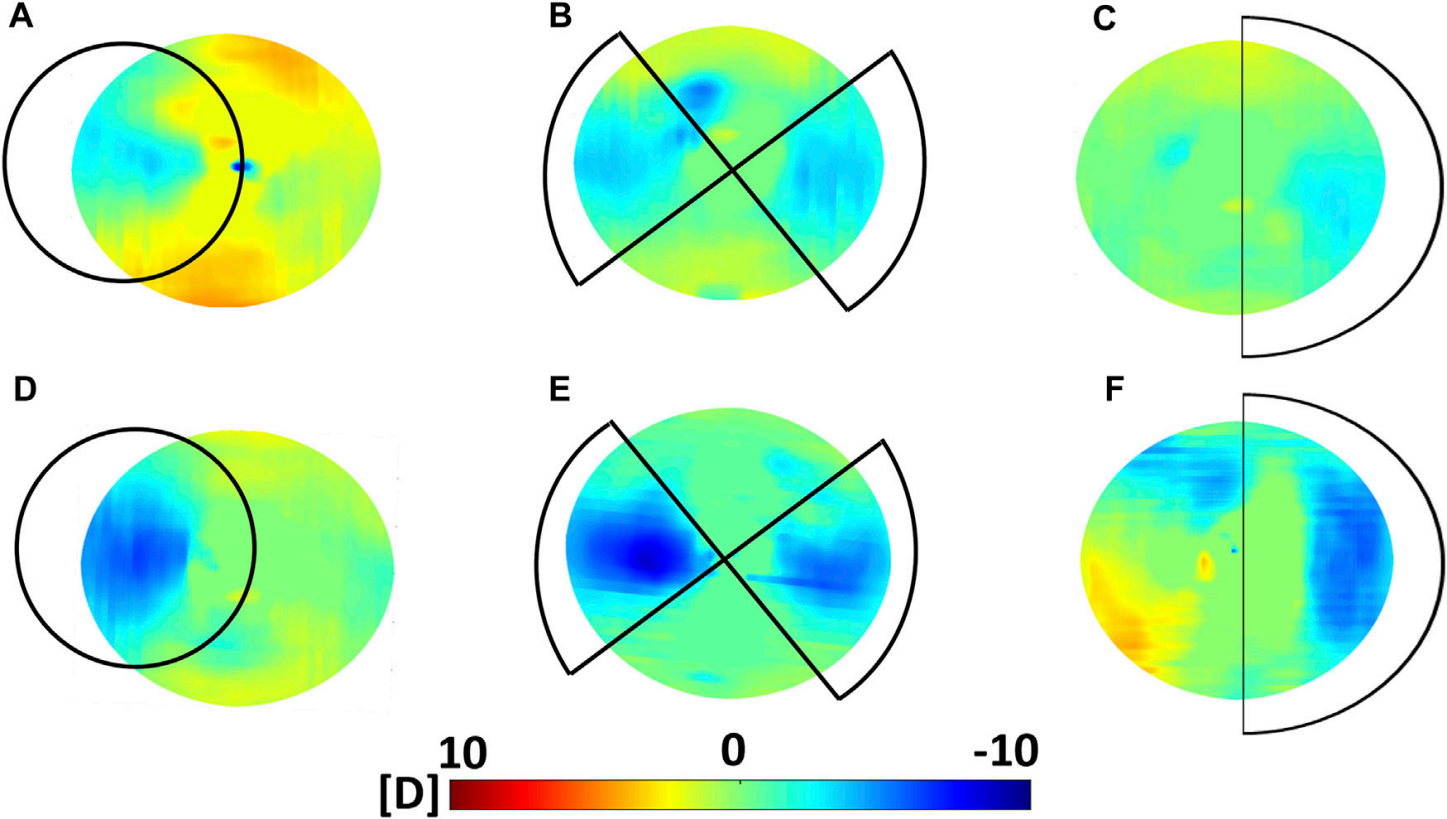
Optical effects induced by CXL in Group A (Dresden Protocol) on the anterior corneal surface. Top panel: variations in sagittal curvature w. r.t the non-irradiated condition as computed 30 min after CXL for **(A)** *circle* (placed off-center and it covered an area larger than the 3 mm optical zone reported in the figure), **(B)** *astigmatism* and **(C)** *coma* irradiation patterns. Bottom panel: variations in sagittal curvature w. r.t the non-irradiated condition as computed 36 h after CXL for **(D)** *circle*, **(E)** *astigmatism* and **(F)** *coma* irradiation patterns.

En-face images in Figure 3 and Figure 4 and data collected in Table 1 show the induced axial strain and change in refractive power (averaged value for the first 350 µm) in response to the standard Dresden CXL (group A), either 30 min or 36 h after treatment, for the three different irradiation patterns. Before the application of patterned CXL, the anterior stroma exhibited homogeneous negative axial strain, suggesting that the cornea is compressed in response to eye inflation. The corresponding baseline values given in Supplementary Table S1 for the *coma*, *astigmatism* and *circle* masks showed no significant differences between the three groups before the irradiation (ANOVA, *p* = 0.293). A general change to positive axial strains was registered in the irradiated areas 30 min after CXL for all irradiation patterns, with statistically significantly different strain values when compared to the pre-irradiation—riboflavin-only—condition (*p* < 0.050). The comparison in terms of local increase in axial strain with respect to the non-irradiated region is shown in Table 1 for the *coma*, *astigmatism,* and *circle* patterns. No significant differences were found between the groups in terms of induced increase in axial strain (ANOVA, *p* = 0.095). Masks corresponding to higher order polynomials (trefoil and quatrefoil) produced a less expected outcome, since the change in sagittal curvature occurred across the whole optical zone, rather than at specific locations (Supplementary Figures S1 and Supplementary Figures S2).

**TABLE 1:** OCE-derived optomechanical indexes for the three CXL patterns (Group A, Dresden Protocol) at different stages of the CXL treatment. Axial strain and sagittal curvature data are reported as differences between the regions irradiated with UVA light w.r.t the non-irradiated ones, with *p*-values referring to the comparison between irradiated and non-irradiated region. *Cyl* values are reported as differences between the pre-CXL condition, with *p*-values referring to the comparison between pre and CXL condition. Due to the compressive nature (=negative sign) of the induced deformation in response to the vacuum, the more positive the difference between strains in the irradiated regions *versus* non-irradiated regions are, the stronger is the mechanical effect of CXL.

		Coma				Astigmatism				Circle			
	*Comparison*	*After CXL*	*p-value*	*36h After CXL*	*p-value*	*After CXL*	*p-value*	*36h After CXL*	*p-value*	*After CXL*	*p-value*	*36h After CXL*	*p-value*
**Axial Strain** [‰]	intra specimen	1.4 ± 0.2	**0.007**	0.6 ± 0.1	**0.019**	2.2 ± 1.1	**0.050**	1.0 ± 0.6	0.082	3.2 ± 0.7	**0.018**	1.1 ± 1.0	0.094
**Sagittal Curvature** [D]	intra specimen	−1.2 ± 0.5	**0.045**	−4.2 ± 1.7	0.051	−2.1 ± 0.8	**0.046**	−4.1 ± 1.5	**0.044**	−4.6 ± 1.4	**0.031**	−4.1 ± 0.8	**0.013**
***Cyl*** [D]	inter specimen	3.3 ± 1.1	0.213	4.4 ± 2.9	0.116	4.1 ± 1.5	**0.041**	6.6 ± 1.9	**0.026**	2.8 ± 2.5	0.195	1.8 ± 2.2	0.292

Bold values indicate statistical significance.

36 h after treatment, the irradiated regions showed less axial strain than the non-irradiated ones when subjected to the same Δp (Table 1), indicating a relatively stiffer behavior. The non-irradiated regions showed compressive strains, while strain in the irradiated areas was close to zero in all three patterns. Again, the variations in axial strain between irradiated and non-irradiated regions were statistically similar among the three groups (ANOVA, *p* = 0.871).

Refractive power analysis showed an overall flattening in response to the patterned CXL treatment. Before CXL treatment, sagittal curvature was approximately constant within an optical zone of 3 mm radius (ANOVA, *p* = 0.521). After treatment, the area irradiated with the *coma* pattern showed a reduction of −1.2 ± 0.5 D compared to the non-irradiated region (*p* = 0.045). A greater reduction in sagittal curvature (−2.1 ± 0.8 D) was observed in the regions irradiated with the *astigmatism* pattern (*p* = 0.046). The induced astigmatic effect induced by CXL was confirmed by the significant increase in the *Cyl* value derived from the Zernike polynomials (*p* = 0.041). The *circle* pattern appeared to induce the highest flattening in terms of sagittal curvature (−4.6 ± 1.4 D), with absolute values ranging from 36.8 ± 2.0 D in the irradiated areas to 41.4 ± 2.8 D (*p* = 0.031) in the non-irradiated areas. In all three subgroups, the regions not affected by CXL displayed non-significant changes in sagittal curvature (*p* > 0.214) compared to the pre-CXL condition, although a slight increase of 1 D in anterior curvature was recorded.

Contrarily, at 36 h after CXL treatment, the corneas showed a uniform decrease in sagittal curvature compared to the preoperative situation, with an overall corneal flattening of 0.9 ± 0.7 D. However, differences between irradiated and non-irradiated regions were still observed and showed greater variation for the *coma* and *astigmatism* pattern compared those measured 30 min after CXL (Table 1). No statistically significant difference was observed between the different patterns 36 h after the treatment (ANOVA, *p* = 0.996).

### The effect of different UV irradiation protocols

**FIGURE 5:**
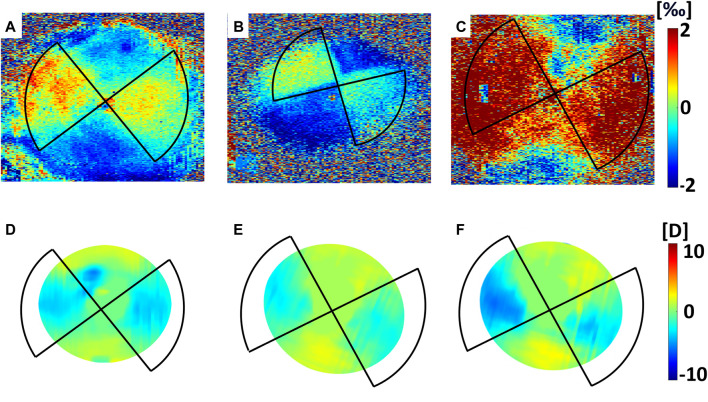
Optomechanical effects induced by CXL in Groups A, B and **(C)**. Top panel: axial strain averaged on the first 350 µm as computed 30 min after CXL in **(A)** Group A (30 min, 3 mW/cm^2^); **(B)** Group B (10 min, 9 mW/cm^2^); **(C)** Group C (30 min, 9 mW/cm^2^). Bottom panel: variations in sagittal curvature w. r.t the non-irradiated condition in **(D)** Group A (30 min, 3 mW/cm^2^); **(E)** Group B (10 min, 9 mW/cm^2^); **(F)** Group C (30 min, 9 mW/cm^2^).

The *astigmatism* CXL pattern was chosen to investigate the effect of three different UV irradiation protocols (Figure 5; Table 2). Similar to group A, the irradiated areas in groups B and C showed a shift toward positive strain in the anterior stroma and an overall flattening of the corneal surface at both 30 min and 36 h after treatment. At 30 min after CXL treatment, the increase in axial strain experienced by group C was higher than in the other two groups (ANOVA *p* = 0.021), with multiple comparison analysis showing significant differences between group C compared to groups A (*post hoc p* = 0.045) and B (*post hoc p* = 0.024). No significant differences were found when comparing groups A and B (*post hoc p* = 0.856). Correspondingly, a trend toward a larger decrease in sagittal curvature in the CXL treated region compared with the non-irradiated area was observed in group C with high-fluence CXL (3.4 ± 0.9 D), when compared to groups A and B (2.1 ± 0.8 D and 2.3 ± 0.6 D, respectively), but did not reach statistical significance (ANOVA *p* = 0.160).

**TABLE 2:** OCE-derived optomechanical indexes for the three different irradiation schemes (*astigmatism* pattern) at different stages of the CXL treatment. Axial strain and sagittal curvature data are reported as differences between the regions irradiated with UVA light w.r.t the non-irradiated ones, with *p*-values referring to the comparison between irradiated and non-irradiated region. *Cyl* values are reported as differences between the pre-CXL condition, with *p*-values referring to the comparison between pre and CXL condition. Due to the compressive nature (=negative sign) of the induced deformation in response to the vacuum, the more positive the difference between strains in the irradiated regions *versus* non-irradiated regions are, the stronger is the mechanical effect of CXL.

		Group A (30 min 3 mW/cm^2^)				Group B (10 min 9 mW/cm^2^)				Group C (30 min 9 mW/cm^2^)			
	*Comparison*	*After CXL*	*p-value*	*36h After CXL*	*p-value*	*After CXL*	*p-value*	*36h After CXL*	*p-value*	*After CXL*	*p-value*	*36h After CXL*	*p-value*
**Axial Strain** [‰]	intra specimen	2.2 ± 1.1	**0.050**	1.0 ± 0.6	0.082	1.4 ± 0.7	0.078	0.6 ± 0.3	0.086	6.9 ± 1.9	**0.025**	1.0 ± 0.3	**0.036**
**Sagittal Curvature** [D]	intra specimen	−2.1 ± 0.8	**0.046**	−4.1 ± 1.5	**0.044**	−2.3 ± 06	**0.021**	−5.0 ± 1.6	**0.032**	−3.4 ± 0.9	**0.023**	−3.7 ± 2.1	0.096
***Cyl*** [D]	inter specimen	4.1 ± 1.5	**0.041**	6.6 ± 1.9	**0.026**	5.0 ± 1.3	**0.023**	6.5 ± 2.3	**0.040**	9.9 ± 1.2	**0.005**	10.7 ± 2.2	**0.015**

Bold values indicate statistical significance.

Regarding induced astigmatism, at 30 min after irradiation ANOVA analysis showed significant differences in *Cyl* values between the groups (*p* = 0.019). There was a significant reduction in all groups compared to the pre-operative condition (*p* ≤ 0.040). In group C, the induced astigmatism was almost two times the amount induced in groups A and B (Table 2).

36 h after irradiation, the differences between irradiated and non-irradiated regions persisted in each of the three groups, with each group showing more positive axial strain and lower sagittal curvature (Table 2) in the irradiated regions.

### The effect of CXL 30 min *versus* 36 h post treatment

The effect of 36 h of tissue storage already had an effect on the measurements, manifested by a 1.7 times lower strain amplitude in the non-irradiated regions compared to 30 min post-operative condition. Regardless of the CXL protocol and irradiation patterns, we observed a difference between the 30 min post-operative and the 36 h post-operative measurements. Central corneal thickness differed (ANOVA *p* < 0.001) when measured after riboflavin instillation (942 ± 70 µm), 30 min after CXL (985 ± 58 µm), and 36 h post-op (1,170 ± 73 µm), with multiple comparison analysis showing significant statistical differences between all groups (*p* < 0.039). 30 min post-CXL, the positive sign of the induced strain in the treated regions indicates axial tissue expansion. In contrast, 36 h after the procedure, a near zero axial strain magnitude in the treated regions indicates very little deformation. The non-irradiated regions showed a compressive behavior, both 30 min (−1.2‰ to −0.7‰) and 36 h after CXL treatment (−0.7‰ to −0.3‰). Accordingly, 30 min after CXL, the difference in terms of axial strain between treated and non-treated regions was almost 3 times larger (1.4‰–3.2‰) than 36 h after the treatment (0.6‰–1.0‰). Therefore, a greater mechanical difference was observed between the irradiated and non-irradiated regions after 36 h after treatment when the eye was inflated. As a result, 36 h after CXL treatment, a 4D higher refractive correction was generally measured in the astigmatism group than 30 min after treatment.

## Discussion

Currently, refractive changes in response to CXL treatment are not considered in the pre-operative planning of keratoconus patients. For the first time, we have quantified the relationship between mechanical and refractive changes in the cornea in response to localized CXL treatment. We demonstrate the potential of using Zernike-based irradiation patterns to enable patient-specific treatment, and highlight the possibility of using the UV fluence to control the extent of stiffening. Finally, we observed substantial differences between CXL effect 30 min after surgery and 36 h after surgery, which may be relevant when interpreting previous *ex vivo* studies on the effects of CXL.

The current study demonstrates that localized CXL treatment has the potential to restrict flattening to certain regions of the cornea and that higher fluences induce a higher degree of flattening. These two parameters could be independently tuned, similarly to the current clinical trend of customized CXL treatment.

The applied non-contact inflation protocol was fast and, being completely noninvasive, it allowed for repeated assessments in the same eye at different time points. The efficacy of the presented OCE setup in capturing the stiffening effect induced by CXL in rat eyes has been proven in a previous study (Kling, 2020). The same protocol has now been adapted to porcine eyes, which are similar to the human in terms of geometry. The assessment of the refractive power has also been included in the acquisition setup. *Ex vivo* tissue is affected by various factors that do not occur *in vivo*, such as swelling, dehydration, endothelial cell death, which could contribute to changes in the cornea geometry. The fact that we applied localized CXL allows an intra-specimen sham control and thus to adequately account for these incidental effects.

Photorefractive intrastromal cross-linking (PiXL) (Lim et al., 2017) has already been shown to achieve refractive changes in the order of −1.62 D with a central spot pattern and high UV fluences (up to 15 J/cm^2^). Our study not only confirmed that mechanical changes are associated with optical changes, but also quantified their relationship for the first time in an experimental setting. In terms of sagittal curvature, we observed a flattening between −1.1 D and −4.6 D in the treated areas. The *astigmatism* pattern induced an increase in *Cyl* value by +3.8 D at 30 min after treatment, which increased to +6.5 D 36 h after the treatment. For comparison, a previous numerical simulation performed on 10 patients with corneal astigmatism predicted a change between −0.74 and −1.23 D for an astigmatic irradiation pattern (Seven et al., 2014). When the UV fluence used for CXL was tripled, we measured a *Cyl* value of +11.4 D, which was almost double the value obtained with standard fluences of 5.4 J/cm^2^ (+6.9 to +7.4 D). These results suggest that triplicating the UV fluence almost doubled the induced refractive correction. Overall, the refractive changes reported in the current study are higher than the ranges described previously both in human studies (Seiler et al., 2016; Elling et al., 2018) where corrections of approximately 1 D was reported, and *in silico*, with a predicted flattening effect of up to 2-3D (Sinha Roy et al., 2013). One could speculate that the differences in the collagen fibers microstructure between human and porcine corneas (Hayes et al., 2007) could result in a different response to the CXL procedure, with the porcine tissue being more susceptible to geometric changes. Another plausible explanation could be that the *ex vivo* tissue used here was compromised by *postmortem* degradation and tissue hydration.

The three irradiation patterns examined induced a similar mechanical strain in the irradiated areas when the same CXL protocol was used, suggesting that the differences between the three patterns result were due solely to the different locations at which CXL was performed.

**FIGURE 6:**
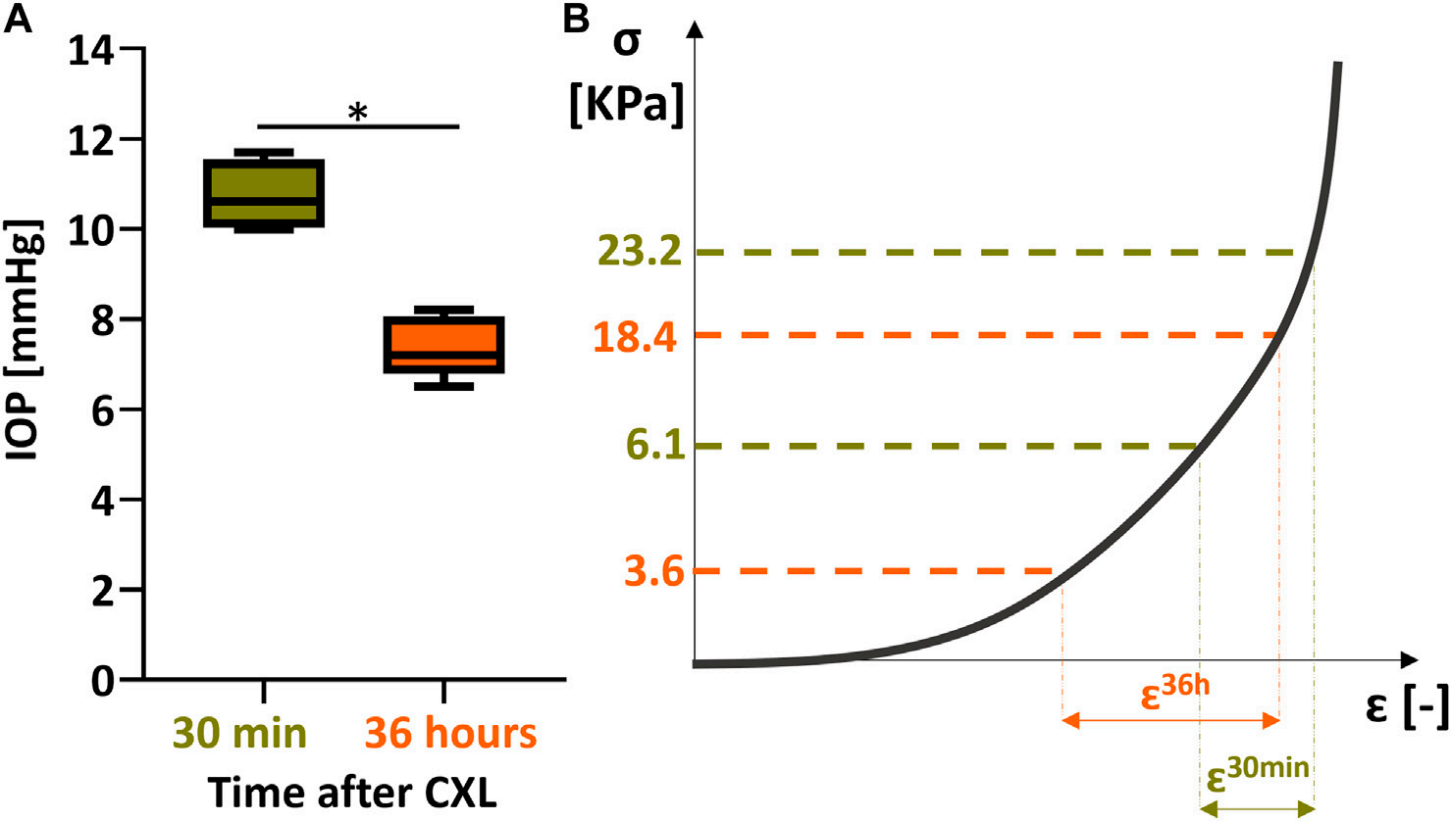
impact of IOP decrease on stiffness computation 36 h after CXL. **(A)** decrease in IOP as measured in a control group of 5 fresh eyes vs. 5 eyes measured after 36 h storage. **(B)** stiffness evaluation 30 min vs. 30 h after CXL.

The immediate mechanical effect induced 30 min after the CXL procedure manifests as a positive axial strain in the irradiated areas, which indicates tissue swelling or relaxation. A similar behavior has been previously described in rat eyes (Kling, 2020). However, this behavior is contradictory to the general understanding of the corneal material properties, which are usually described and modeled as a hyperelastic material that exhibits lateral contraction during elongation. According, one would expect that a stiffer region to experience less axial compression during inflation than the surrounding non-irradiated areas. This is the situation we encountered 36 h after the treatment where the irradiated regions showed a stiffer behavior than the non-irradiated surrounding. Even though bending could be an alternative response of the corneal tissue in response to CXL, we can rule out its presence by the fact that this study measures the mechanical axial strain rather than displacement, showing directly tissue compression or relaxation. This discrepancy between the measurements at 30 min and 36 h leads us to hypothesize that the immediate effect observed in response to CXL treatment could be the result of a change in tissue hydration corresponding to dehydration of the anterior surface, or the result of osmotic pressure created by the newly created cross-links within the cornea. At 36 h after treatment, the corneal hydration had time to equilibrate, so that the permanent mechanical effect of CXL can be better isolated. Despite the fact that the eyes were stored in a Dextran 5% solution, corneal swelling was observed 36 h after the treatment. This effect can be explained by the tissue striving to reach its osmotic equilibrium, which has been modified both due to the de-epithelialization and the CXL treatment. At the same time, the IOP decreased in the post-mortem eyes, which due to a reduced stress on the ocular wall similarly can favor an increase in thickness. After 36 h, the difference in axial strain between treated and non-treated regions was smaller than 30 min after CXL treatment. On the one hand, irradiated zones of the cornea swelled less than non-irradiated zones during the preservation time, resulting in a different thickness between the internal control regions. On the other hand, the IOP would naturally decrease during the 36 h preservation period, shifting the measurement point towards the left on the non-linear stress-strain curve (where a hyperelastic tissue such as the cornea becomes weaker). To quantify this effect, we invasively measured the IOP in 10 pig eyes: 5 eyeballs were tested within 12 h of collection and 5 eyeballs 36 h after preservation in a MeM+5% Dextran solution, showing a pressure drop from 10.7 mmHg to 7.4 mmHg (*p* = 0.016, Figure 6A). For an approximation of the stress in the tissue before and after storage, the Laplace law was applied. Under the hypothesis of a thin wall, the tangential stress is defined as $\sigma_{t}∼\frac{IOP*r}{2*t}$ , where *r =* $\frac{1.3375-1}{K}$ is the anterior radius and *t* the experimentally determined mean thickness of the cornea. Considering the measured IOP and corneal thickness, the stress in fresh corneas is approximately σ_t_ = 6.1 kPa, and 36 h after collection it decreases to approximately σ_t_ = 3.6 kPa. As a result, the tissue is subjected to lower stress prior testing, which sets it on a different position on the nonlinear stress-strain curve (Figure 6B). In addition, modulation of the ambient pressure with a Δp of 30 mmHg during measurement resulted in an applied Δσ_t_ of 17.1 kPa in fresh corneas and of 14.8 kPa in 36-h preserved corneas. In our experiment, strains in the non-irradiated region were 1.7x smaller at 36 h post-op compared to 30 min after CXL, which is in contrast to our expectation that the tissue would behave softer at lower pre-stress values (Figure 6B), but could be partially explained by the reduced stress applied after 36 h storage. To fully understand the reason for the reduced response in terms of axial strain, these two effects (thickness increase and pressure decrease) should be decoupled and investigated separately. In view of the uncertainty these variables add, the value of the current study lies in the fact that each sample presents an internal control and the same pre-op thickness, which allowed to assess the effect of CXL even in the presence of swelling and IOP decrease.

In all three irradiation patterns, we observed good agreement between regions that 1) were irradiated, 2) showed a shift toward positive strains, and 3) presented a flattening in sagittal curvature (Figure 3; Figure 4). Interestingly, a trend towards a slight increase in optical power was measured in the areas outside the treated regions, which could be a direct consequence of defining sagittal curvature from the deformed OCT scan. It should be noted that the good agreement between optical and mechanical changes in the cornea is limited to irradiation patterns corresponding to low-order Zernike polynomials, whereas masks inspired by higher-order polynomials yielded a less predictable result in terms of refractive changes, which were not only restricted to the irradiated areas. This observation could be related to the fact that these masks did not perfectly shield the light from the non-irradiated areas, resulting to the CXL stiffening also extending peripherally to the irradiated area. In contrast to laser refractive surgery, patterned CXL treatment effectively is indeed a more indirect approach for inducing refractive changes, but at the same time promises to be more conservative. Therefore, the observed joint optomechanical response is a valuable input for future numerical simulations aimed at predicting and personalizing photorefractive CXL treatment.

Our results confirm that increasing the administered UV fluence (energy) during treatment result in a more pronounced mechanical stiffening and refractive correction (Figure 5). Three different CXL protocols were tested: Standard Dresden protocol, accelerated CXL (both with a fluence of 5.4 J/cm^2^) and a high-fluence protocol with 16 J/cm^2^. The latter is three times the energy applied in standard Dresden CXL, but similar to the 15 J/cm^2^ protocol used in photorefractive intrastromal CXL (Elling et al., 2018). With the increase in UV fluence delivered to the human corneas during CXL, the risk of damaging endothelial cells must be seriously considered. As described by Seiler and others, the corneal endothelium can tolerate significantly more irradiation damage than previously thought (Seiler et al., 2019). The safe upper limit of total fluence delivered during treatment is therefore higher than the limit of 5.4 J/cm^2^ originally established (Wollensak et al., 2003) when the Dresden protocol was introduced for the treatment of keratoconus.

30 min after treatment, the high-fluence CXL had significantly higher axial strain amplitude compared with other CXL protocols, while no differences were found between the two standard-fluence groups. The latter finding is consistent with clinical practice, according to which the 9 mW-5.4 J/cm^2^ accelerated CXL protocols is considered equivalent (Mazzotta et al., 2021), and confirms the results of an experimental study suggesting equivalency of accelerated protocols up to 45 mW/cm^2^ (Wernli et al., 2013). We found that not only mechanical, but also the refractive correction induced by these two 5.4 J/cm^2^ treatments was comparable, confirming the *in vivo* observations of Lang et al., that maximal keratometry and corrected visual acuity was comparable in CXL groups treated with 3 mW/cm^2^ for 30 min and 9 mW/cm^2^ for 10 min (Lang et al., 2019).

The current study is not free of limitations, the most important one being a small sample size, which does not allow for robust statistical analyses. Despite this limitation, the results were clear and confirm our hypotheses. The current sample size allowed us to sense detectable statistically significant changes of 1.6‰ in axial strain and a corresponding 3 D decrease in sagittal curvature with an α error of 0.05 and a power of 80%. The sample size was therefore interpreted as large enough to confirm the hypotheses underlying this study. Another limitation is the use of *ex vivo* samples, which are subject to changes in hydration and post-mortem degradation. Although precautions were taken to minimize these effects (use of fresh samples, preservation media to avoid swelling), we could not completely eliminate them. Because of the high sensitivity of OCE to small deformations, the proposed technique is also susceptible to side effects such as deformations resulting from small changes in tissue hydration such as induced by repeated dripping of riboflavin solution. Future studies are needed to evaluate these potentially co-occurring dynamic effects. The temperature in the laboratory room was not actively controlled while the experiments were performed, with small changes in this parameter potentially affecting the hydration level of the corneas. Lastly, the present experimental setup was incompatible with an invasive pressure measurement, which would have prevented OCE measurements within the pressure chamber. Given that an ultrasound pachymeter was not available, IOP was assumed constant across all the sample. The validity of this assumption was verified invasively measuring IOP on 5 separate corneas, which showed values of 10.7 ± 0.8 mmHg.

In conclusion, the main findings of the present study are as follows: i) OCE allows high-resolution visualization and quantification of the optomechanical effects of CXL in the anterior stroma; ii) mechanical stiffening is accompanied by geometric and thus refractive changes in the cornea; iii) tripling the energy delivered during CXL nearly doubles the induced refractive correction; iv) cylindrical refractive changes of 2–4 D can be achieved with patterned irradiation and a standard CXL protocol.

## Data Availability

The raw data supporting the conclusion of this article will be made available by the authors, without undue reservation.

## References

[B1] Abrishamchi R. Abdshahzadeh H. Hillen M. Hafezi N. Torres-Netto E. A. Aslanides I. M. (2021). High-fluence accelerated epithelium-off corneal cross-linking protocol provides dresden protocol–like corneal strengthening. Transl. Vis. Sci. Technol. 10 (5), 10–17. 10.1167/tvst.10.5.10 PMC8458988 34542574

[B2] Ambrósio R. Lopes B. T. Faria-Correia F. Salomão M. Q. Bühren J. Roberts C. J. (2017). Integration of scheimpflug-based corneal tomography and biomechanical assessments for enhancing ectasia detection. J. Refract Surg. 33 (7), 434–443. 10.3928/1081597x-20170426-02 28681902

[B3] Ariza-Gracia M. Á Zurita J. F. Piñero D. P. Rodriguez-Matas J. F. Calvo B. (2015). Coupled biomechanical response of the cornea assessed by non-contact tonometry. A simulation study. PLoS One 10 (3), 01214866. 10.1371/journal.pone.0121486 PMC4364121 25780915

[B4] Boschetti F. Conti D. Soriano E. M. Mazzotta C. Pandolfi A. (2021). Experimental *in-vitro* investigation on Epi-Off-Crosslinking on porcine corneas. PLoS One 16 (4), 02499499. 10.1371/journal.pone.0249949 PMC8049322 33857213

[B5] Curatolo A. Birkenfeld J. S. Martinez-Enriquez E. Germann J. A. Muralidharan G. Palací J. (2020). Multi-meridian corneal imaging of air-puff induced deformation for improved detection of biomechanical abnormalities. Biomed. Opt. Express 11 (11), 6337. 10.1364/boe.402402 33282494 PMC7687933

[B6] De Stefano V. S. Ford M. R. Seven I. Dupps W. J. (2020). Depth-dependent corneal biomechanical properties in normal and keratoconic subjects by optical coherence elastography. Transl. Vis. Sci. Technol. 9 (7), 4–10. 10.1167/tvst.9.7.4 PMC7414661 32832211

[B7] Elling M. Kersten-Gomez I. Dick H. B. (2018). Photorefractive intrastromal corneal crosslinking for treatment of myopic refractive error: findings from 12-month prospective study using an epithelium-off protocol. J. Cataract. Refract Surg 44 (4), 487–495. Available from. 10.1016/j.jcrs.2018.01.022 29778107

[B8] Esporcatte L. P. G. Salomão M. Q. Lopes B. T. Vinciguerra P. Vinciguerra R. Roberts C. (2020). Biomechanical diagnostics of the cornea. Eye Vis. 7 (1), 9–12. 10.1186/s40662-020-0174-x PMC7001259 32042837

[B9] Hayes S. Boote C. Kamma-Lorger C. S. Rajan M. S. Harris J. Dooley E. (2011). Riboflavin/UVA collagen cross-linking-induced changes in normal and keratoconus corneal stroma. PLoS One 6 (8), e22405–e22409. 10.1371/journal.pone.0022405 21850225 PMC3151245

[B10] Hayes S. Boote C. Lewis J. Sheppard J. Abahussin M. Quantock A. J. (2007). Comparative study of fibrillar collagen arrangement in the corneas of primates and other mammals. Anat. Rec. 290 (12), 1542–1550. 10.1002/ar.20613 17957749

[B11] Hersh P. S. Greenstein S. A. Fry K. L. (2011). Corneal collagen crosslinking for keratoconus and corneal ectasia: one-year results. J. Cataract. Refract Surg 37 (1), 149–160. Available from. 10.1016/j.jcrs.2010.07.030 21183110

[B12] Hong J. Xu J. Wei A. Deng S. X. Cui X. Yu X. (2013). A new tonometer-the corvis ST tonometer: clinical comparison with noncontact and goldmann applanation tonometers. Investig. Ophthalmol. Vis. Sci. 54 (1), 659–665. 10.1167/iovs.12-10984 23307970

[B13] Kennedy B. F. Koh S. H. McLaughlin R. A. Kennedy K. M. Munro P. R. T. Sampson D. D. (2012). Strain estimation in phase-sensitive optical coherence elastography. Biomed. Opt. Express 3 (8), 1865. 10.1364/boe.3.001865 22876350 PMC3409705

[B14] Kirby M. A. Pelivanov I. Regnault G. Pitre J. J. Wallace R. T. O’Donnell M. (2023). Acoustic micro-tapping optical coherence elastography to quantify corneal collagen cross-linking: an *ex vivo* human study. Ophthalmol. Sci 3 (2), 100257. Available from:. 10.1016/j.xops.2022.100257 36685713 PMC9852959

[B15] Kling S. (2020). Optical coherence elastography by ambient pressure modulation for high-resolution strain mapping applied to patterned cross-linking. J. R. Soc. Interface 17 (162), 20190786. 10.1098/rsif.2019.0786 31964268 PMC7014812

[B16] Kling S. (2021). *In-vivo* measurement of ocular deformation in response to ambient pressure modulation. Front. Bioeng. Biotechnol. 9 (11), 759588–8. 10.3389/fbioe.2021.759588 34869269 PMC8634479

[B17] Kling S. Akca I. B. Chang E. W. Scarcelli G. Bekesi N. Yun S. H. (2014). Numerical model of optical coherence tomographic vibrography imaging to estimate corneal biomechanical properties. J. R. Soc. Interface 11 (101), 20140920. 10.1098/rsif.2014.0920 25320067 PMC4223913

[B18] Kling S. Khodadadi H. Goksel O. (2020). Optical coherence elastography-based corneal strain imaging during low-amplitude intraocular pressure modulation. Front. Bioeng. Biotechnol. 7, 453. 10.3389/fbioe.2019.00453 32083064 PMC7004960

[B19] Kling S. Marcos S. (2013). Contributing factors to corneal deformation in air puff measurements. Investig. Ophthalmol. Vis. Sci. 54 (7), 5078–5085. 10.1167/iovs.13-12509 23821200

[B20] Kwok S. J. J. Kuznetsov I. A. Kim M. Choi M. Scarcelli G. Yun S. H. (2016). Selective two-photon collagen crosslinking *in situ* measured by Brillouin microscopy. Optica 3 (5), 469–472. 10.1364/optica.3.000469 28983498 PMC5626012

[B21] Lan G. Aglyamov S. R. Larin K. V. Twa M. D. (2021). *In vivo* human corneal shear-wave optical coherence elastography. Optom. Vis. Sci. 98 (1), 58–63. 10.1097/opx.0000000000001633 33394932 PMC7774819

[B22] Lang P. Z. Hafezi N. L. Khandelwal S. S. Torres-netto E. A. Hafezi F. Randleman J. B. (2019). Comparative functional outcomes after corneal crosslinking using standard, accelerated, and accelerated with higher total fluence protocols. Cornea 38 (4), 433–441. 10.1097/ico.0000000000001878 30681515

[B23] Larin K. V. Sampson D. D. (2017). Optical coherence elastography – OCT at work in tissue biomechanics [Invited]. Biomed. Opt. Express 8 (2), 1172. 10.1364/boe.8.001172 28271011 PMC5330567

[B24] Lim W. K. Da Soh Z. Choi H. K. Y. Theng Jts (2017). Epithelium-on photorefractive intrastromal cross-linking (PiXL) for reduction of low myopia. Clin. Ophthalmol. 11, 1205–1211. 10.2147/opth.s137712 28721004 PMC5499923

[B25] Liu T. Shen M. Li H. Zhang Y. Mu B. Zhao X. (2020). Changes and quantitative characterization of hyper-viscoelastic biomechanical properties for young corneal stroma after standard corneal cross-linking treatment with different ultraviolet-A energies. Acta Biomater. 113, 438–451. 10.1016/j.actbio.2020.06.005 32525050

[B26] Matveyev A. L. Matveev L. A. Sovetsky A. A. Gelikonov G. V. Moiseev A. A. Zaitsev V. Y. (2018). Vector method for strain estimation in phase-sensitive optical coherence elastography. Laser Phys. Lett. 15 (6), 065603. 10.1088/1612-202x/aab5e9

[B27] Mazzotta C. Raiskup F. Hafezi F. Torres-Netto E. A. Armia Balamoun A. Giannaccare G. (2021). Long term results of accelerated 9 mW corneal crosslinking for early progressive keratoconus: the Siena Eye-Cross Study 2. Eye Vis. 8 (1), 16–12. 10.1186/s40662-021-00240-8 PMC8088009 33931101

[B28] McAuley R. Nolan A. Curatolo A. Alexandrov S. Zvietcovich F. Varea Bejar A. (2022). Co-axial acoustic-based optical coherence vibrometry probe for the quantification of resonance frequency modes in ocular tissue. Sci. Rep. 12, 18834. 10.1038/s41598-022-21978-8 36336702 PMC9637745

[B29] Nair A. Singh M. Aglyamov S. Larin K. V. (2021). Heartbeat optical coherence elastography: corneal biomechanics *in vivo* . J. Biomed. Opt. 26 (02), 020502–020508. 10.1117/1.jbo.26.2.020502 33624461 PMC7901857

[B30] Ramier A. Eltony A. M. Chen Y. T. Clouser F. Birkenfeld J. S. Watts A. (2020). *In vivo* measurement of shear modulus of the human cornea using optical coherence elastography. Sci. Rep. 10 (1), 17366. 10.1038/s41598-020-74383-4 33060714 PMC7567833

[B31] Richoz O. Hammer A. Tabibian D. Gatzioufas Z. Hafezi F. (2013). The biomechanical effect of corneal collagen cross-linking (CXL) with riboflavin and UV-A is oxygen dependent. Transl. Vis. Sci. Technol. 2 (7), 6. 10.1167/tvst.2.7.6 24349884 PMC3860351

[B32] Sachdev G. S. Ramamurthy S. Dandapani R. (2020). Photorefractive intrastromal corneal crosslinking for treatment of low myopia: clinical outcomes using the transepithelial approach with supplemental oxygen. J. Cataract. Refract Surg. 46 (3), 428–433. 10.1097/j.jcrs.0000000000000073 32050206

[B33] Sanchez I. Martin R. Ussa F. Fernandez-Bueno I. (2011). The parameters of the porcine eyeball. Graefe’s Arch. Clin. Exp. Ophthalmol. 249 (4), 475–482. 10.1007/s00417-011-1617-9 21287191

[B34] Scarcelli G. Kling S. Quijano E. Pineda R. Marcos S. Yun S. H. (2013). Brillouin microscopy of collagen crosslinking: noncontact depth-dependent analysis of corneal elastic modulus. Investig. Ophthalmol. Vis. Sci. 54 (2), 1418–1425. 10.1167/iovs.12-11387 23361513 PMC3597196

[B35] Scarcelli G. Yun S. H. (2007). Confocal Brillouin microscopy for three-dimensional mechanical imaging. Nat. Photonics 2 (2), 39–43. 10.1038/nphoton.2007.250 19812712 PMC2757783

[B36] Schmitt J. M. (1998). OCT elastography: imaging microscopic deformation and strain of tissue. Opt. Express 3 (6), 199. 10.1364/oe.3.000199 19384362

[B37] Seiler T. G. Batista A. Frueh B. E. Koenig K. (2019). Riboflavin concentrations at the endothelium during corneal cross-linking in humans. Investig. Ophthalmol. Vis. Sci. 60 (6), 2140–2145. 10.1167/iovs.19-26686 31099830

[B38] Seiler T. G. Fischinger I. Koller T. Zapp D. Frueh B. E. Seiler T. (2016). Customized corneal cross-linking: one-year results. Am. J. Ophthalmol. 166 (1), 14–21. 10.1016/j.ajo.2016.02.029 26944278

[B39] Seven I. Roy A. S. Dupps W. J. (2014). Patterned corneal collagen crosslinking for astigmatism: computational modeling study. J. Cataract. Refract Surg. 40 (6), 943–953. Available from. 10.1016/j.jcrs.2014.03.019 24767795 PMC4062190

[B40] Shao P. Eltony A. M. Seiler T. G. Tavakol B. Pineda R. Koller T. (2018). Spatially-resolved Brillouin spectroscopy reveals biomechanical changes in early ectatic corneal disease and post-crosslinking *in vivo* . arXiv Quant Methods.

[B41] Singh M. Li J. Han Z. Raghunathan R. Nair A. Wu C. (2017). Assessing the effects of riboflavin/UV-A crosslinking on porcine corneal mechanical anisotropy with optical coherence elastography. Biomed. Opt. Express 8 (1), 349. 10.1364/boe.8.000349 28101423 PMC5231304

[B42] Sinha Roy A. Rocha K. M. Randleman J. B. Stulting R. D. Dupps W. J. (2013). Inverse computational analysis of invivo corneal elastic modulus change after collagen crosslinking for keratoconus. Exp. Eye Res. 113, 92–104. Available from. 10.1016/j.exer.2013.04.010 23664859 PMC4104483

[B43] Spoerl E. Huhle M. Seiler T. (1998). Induction of cross-links in corneal tissue. Exp. Eye Res. 66 (1), 97–103. 10.1006/exer.1997.0410 9533835

[B44] Stodulka P. Halasova Z. Slovak M. Sramka M. Liska K. Polisensky J. (2020). Photorefractive intrastromal crosslinking for correction of hyperopia: 12-month results. J. Cataract. Refract Surg. 46 (3), 434–440. 10.1097/j.jcrs.0000000000000074 32142499

[B45] Thibos L. N. Xin H. Bradley A. Applegate R. A. (2004). Accuracy and precision of objective refraction from wavefront aberrations. J. Vis. 4 (4), 329–351. 10.1167/4.4.9 15134480

[B46] Vinciguerra R. Ambrósio R. Elsheikh A. Roberts C. J. Lopes B. Morenghi E. (2016). Detection of keratoconus with a new biomechanical index. J. Refract Surg. 32 (12), 803–810. 10.3928/1081597x-20160629-01 27930790

[B47] von F. (1934). Zernike. Beugungstheorie des schneidenver-fahrens und seiner verbesserten form, der phasenkontrastmethode. Phys. 1 (7), 689–704. 10.1016/S0031-8914(34)80259-5

[B48] Wernli J. Schumacher S. Spoerl E. Mrochen M. (2013). The efficacy of corneal cross-linking shows a sudden decrease with very high intensity UV light and short treatment time. Investig. Ophthalmol. Vis. Sci. 54 (2), 1176–1180. 10.1167/iovs.12-11409 23299484

[B49] Wollensak G. Spoerl E. Seiler T. (2003). Riboflavin/ultraviolet-A-induced collagen crosslinking for the treatment of keratoconus. Am. J. Ophthalmol. 135 (5), 620–627. 10.1016/s0002-9394(02)02220-1 12719068

[B50] Zaitsev V. Y. Matveyev A. L. Matveev L. A. Gelikonov G. V. Sovetsky A. A. Vitkin A. (2016). Optimized phase gradient measurements and phase-amplitude interplay in optical coherence elastography. J. Biomed. Opt. 21 (11), 116005. 10.1117/1.jbo.21.11.116005 27824215

[B51] Zhang Y. Conrad A. H. Conrad G. W. (2011). Effects of ultraviolet-A and riboflavin on the interaction of collagen and proteoglycans during corneal cross-linking. J. Biol. Chem. 286 (15), 13011–13022. 10.1074/jbc.M110.169813 21335557 PMC3075647

[B52] Zvietcovich F. Nair A. Ambekar Y. S. Singh M. Aglyamov S. R. Twa M. D. (2020). Confocal air-coupled ultrasonic optical coherence elastography probe for quantitative biomechanics. Opt. Lett. 45 (23), 6567. 10.1364/ol.410593 33258863 PMC10041740

[B53] Zvietcovich F. Nair A. Singh M. Aglyamov S. R. Twa M. D. Larin K. V. (2022). *In vivo* assessment of corneal biomechanics under a localized cross-linking treatment using confocal air-coupled optical coherence elastography. Biomed. Opt. Express 13 (5), 2644–2654. 10.1364/boe.456186 35774330 PMC9203097

[B54] Zykov A. A. Matveyev A. L. Sovetsky A. A. Matveev L. A. Zaitsev V. Y. (2023). Vector method of strain estimation in OCT-elastography with adaptive choice of scale for estimating interframe phase-variation gradients. Laser Phys. Lett. 20 (9), 095601. 10.1088/1612-202x/ace253

