# Supplementary material for: Optomechanical assessment of photorefractive corneal cross-linking via optical coherence elastography
Source: Front Bioeng Biotechnol. 2023 Nov 13;11:1272097. doi: 10.3389/fbioe.2023.1272097 (PMC10680454; doi:10.3389/fbioe.2023.1272097)
Supplement: Supplementary file 1 [file DataSheet1.docx]

Supplementary Material

Optomechanical Assessment of Photorefractive Corneal Cross-linking via Optical Coherence Elastography

Matteo Frigelli, Philippe Büchler, Sabine Kling^*^

*** Correspondence:** Sabine Kling: klings@ee.ethz.ch

# Supplementary Tables

Table S1: OCE-derived optomechanical indexes for the three CXL patterns (Group A, Dresden Protocol) at different stages of the CXL treatment. Axial strain and sagittal curvature data are reported for the regions irradiated with UVA light.

|  | **Coma** | | |  | **Astigmatism** | | |  | **Circle** | | |
| --- | --- | --- | --- | --- | --- | --- | --- | --- | --- | --- | --- |
|  | *Before CXL* | *After CXL* | *36h After CXL* |  | *Before*  *CXL* | *After CXL* | *36h After*  *CXL* |  | *Before*  *CXL* | *After CXL* | *36h After CXL* |
| **Axial Strain** [‰] | -0.7 ± 0.2 | 0.8 ± 0.1 | -0.06 ± 0.6 |  | -1.6 ± 0.8 | 0.6 ± 0.5 | -0.02 ± 0.08 |  | -1.0 ± 0.7 | 2.1 ± 0.5 | 0.08 ± 0.04 |
| **Sagittal Curvature** [D] | 39.5 ± 0.7 | 38.3 ± 0.4 | 35.5 ± 1.2 |  | 40.0 ± 0.7 | 38.1 ± 0.9 | 34.3 ± 0.7 |  | 40.9 ± 2.2 | 36.8 ± 2.0 | 32.6 ± 1.5 |
| ***Cyl*** [D] | -1.9 ± 0.7 | -3.8 ± 2.2 | -6.4 ± 2.3 |  | -2.8 ± 0.2 | -6.9 ± 1.3 | -9.4 ± 1.9 |  | -2.6 ± 0.3 | -5.4 ± 2.9 | -4.5 ± 1.9 |
| **Zernike fit error** [µm] | 6.3 ± 1.6 | 7.3 ± 0.4 | 5.5 ± 1.9 |  | 7.5 ± 3.8 | 10.3 ± 4.2 | 7.4 ± 3.6 |  | 6.4 ± 1.1 | 7.9 ± 3.2 | 5.2 ± 0.6 |

Table S2: OCE-derived optomechanical indexes for the three different irradiation schemes (*astigmatism* pattern) at different stages of the CXL treatment. Axial strain and sagittal curvature data are reported for the regions irradiated with UVA light.

|  | **Group A (30 min 3mW/cm^2^)** | | |  | **Group B (10 min 9mW/cm^2^)** | | |  | **Group C (30 min 9mW/cm^2^)** | | |
| --- | --- | --- | --- | --- | --- | --- | --- | --- | --- | --- | --- |
|  | *Before*  *CXL* | *After CXL* | *36h After*  *CXL* |  | *Before*  *CXL* | *After CXL* | *36h After CXL* |  | *Before*  *CXL* | *After CXL* | *36h After CXL* |
| **Axial Strain** [‰] | -1.6 ± 0.8 | 0.6 ± 0.5 | -0.02 ± 0.08 |  | -1.2 ± 0.6 | 0.2 ± 0.2 | -0.03 ± 0.07 |  | -1.2 ± 0.4 | 5.6 ± 0.2 | 0.09 ± 0.1 |
| **Sagittal Curvature** [D] | 40.0 ± 0.7 | 38.1 ± 0.9 | 34.3 ± 0.7 |  | 39.3 ± 0.9 | 39.6 ± 1.1 | 37.4 ± 1.4 |  | 39.2 ± 0.5 | 38.1 ± 1.4 | 36.0 ± 2.8 |
| ***Cyl*** [D] | -2.8 ± 0.2 | -6.9 ± 1.3 | -9.4 ± 1.9 |  | -2.4 ± 0.6 | -7.4 ± 1.9 | -8.8 ± 2.5 |  | -1.4 ± 0.4 | -11.4 ± 1.1 | -12.1 ± 2.3 |
| **Zernike fit error** [µm] | 7.5 ± 3.8 | 10.3 ± 4.2 | 7.4 ± 3.6 |  | 8.0 ± 5.1 | 7.1 ± 3.9 | 6.7 ± 5.2 |  | 7.3 ± 2.1 | 10.6 ± 2.6 | 8.3 ± 1.4 |

# Supplementary Figures


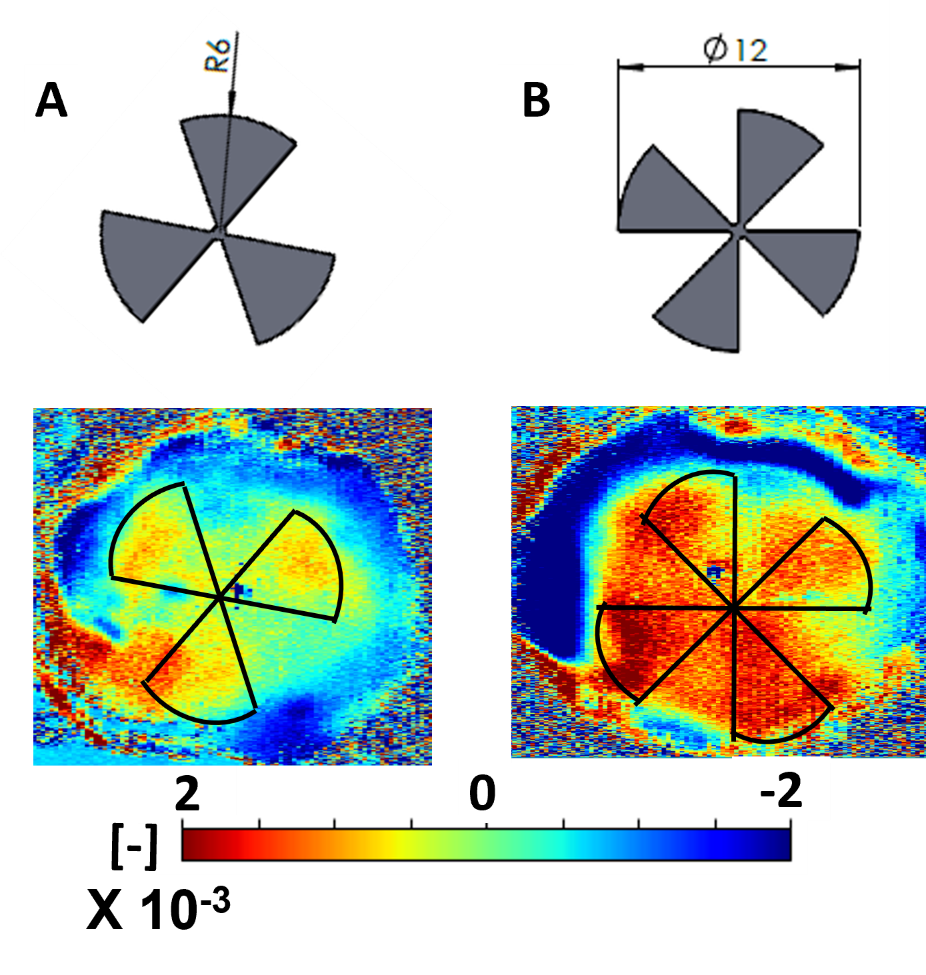


**Figure S1.** Mechanical effects induced by patterned CXL applying higher order polynomials masks. Top panel: mask design. Bottom panel: axial strain as computed 30 min after CXL for (**A**) trefoil and (**B**) quatrefoil patterns.


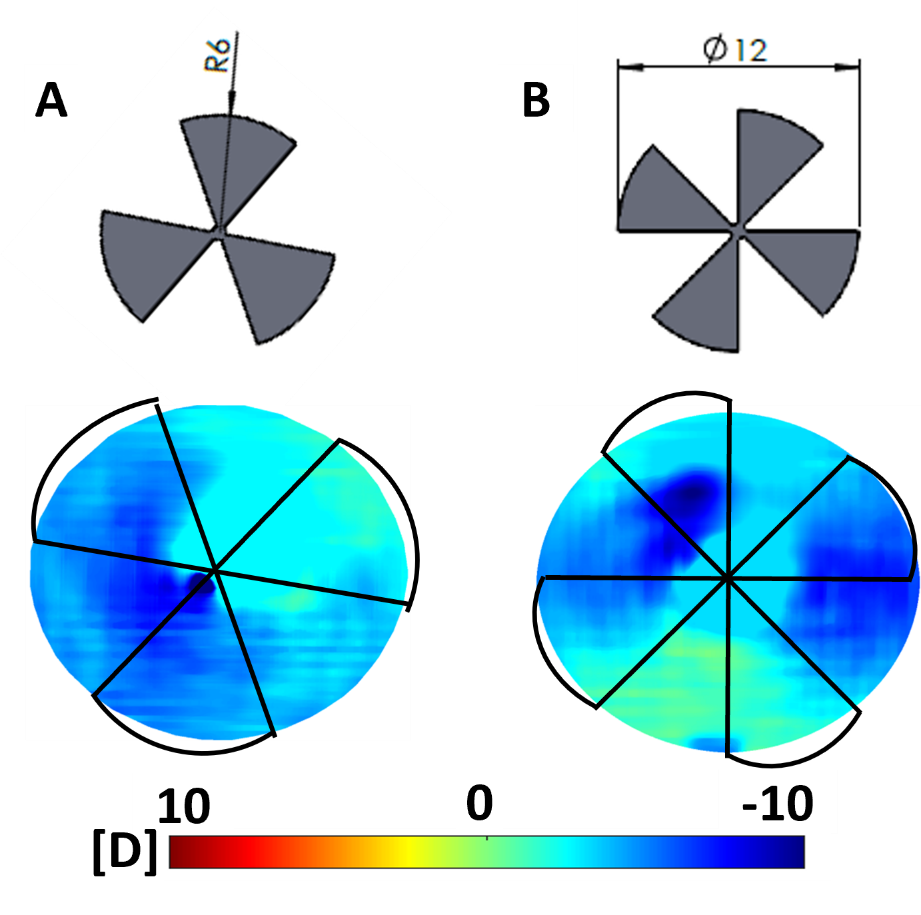


**Figure S2.** Refractive effects induced by patterned CXL applying higher order polynomials masks. Top panel: mask design. Bottom panel: variations in sagittal curvature w.r.t the non-irradiated condition as computed 30 min after CXL for (**A**) trefoil and (**B**) quatrefoil patterns.
